# A Method for Enhancing the Positioning Performance of PPP-B2b by Integrating Galileo Observation

**DOI:** 10.3390/s26103073

**Published:** 2026-05-13

**Authors:** Xuena Shang, Liwenle Liu, Yilong Yuan, Mengxiang Tong, Qianqian He, Xiaopeng Gong

**Affiliations:** 1 School of Geodesy and Geomatics, Wuhan University, 129 Luoyu Road, Wuhan 430079, China; 2023302141058@whu.edu.cn; 2 GNSS Research Center, Wuhan University, 129 Luoyu Road, Wuhan 430079, China; lwlliu@whu.edu.cn (L.L.); xpgong@whu.edu.cn (X.G.); 3 Tencent Technology (Beijing) Co., Ltd., Northwest Wangdong Road, Beijing 100193, China; yosonyuan@tencent.com (Y.Y.); erictong@tencent.com (M.T.); 4 China Electronics Technology Group Corporation No. 15th Research Institute, No. 211 Beisihuan Middle Road, Beijing 100083, China; 5 School of Electronic and Information Engineering, Beihang University, Beijing 100191, China

**Keywords:** precise point positioning, PPP-B2b, Galileo, broadcast ephemeris

## Abstract

**Highlights:**

**What are the main findings?**

- Integration of Galileo observation significantly improves PPP-B2b performance, increasing satellite availability and reducing DOP values.
- GPS/BDS-3/Galileo PPP shortens convergence time by approximately 13–17% horizontally and 18–20% vertically compared with the GPS/BDS-3 solution based on the PPP-B2b service and broadcast ephemeris (68%).

**What are the implications of the main findings?**

- Galileo effectively enhances satellite geometry and serves as a robust complement to the regional PPP-B2b service.
- The integration improves positioning robustness and availability, especially in challenging environments with limited satellite visibility.

**Abstract:**

The BeiDou-3 (BDS-3) Precise Point Positioning service (PPP-B2b) can realize decimeter-level positioning by broadcasting satellite orbit, clock offset, and code bias corrections via GEO satellites, enabling PPP without reliance on ground communication networks. However, the current PPP-B2b service only provides corrections for BDS-3 and GPS satellites, which limits the number of available satellites and may affect positioning performance in challenging environments. To further enhance the positioning performance, we propose to incorporate Galileo observation into the PPP-B2b positioning. A PPP model integrating PPP-B2b service and broadcast ephemeris was established. First, the accuracy of the Galileo broadcast ephemeris was evaluated using precise orbit and clock products as references. The results show that the mean signal-in-space range error (SISRE) standard deviation of Galileo broadcast ephemeris is 0.30, which is only a little worse than that of GPS from PPP-B2b service. Then, the positioning experiments were conducted under different elevation cutoff angles. The experiments were conducted using data from 94 reference stations in China over a 7-day period. The results demonstrate that the inclusion of Galileo satellites significantly increases the number of visible satellites and improves satellite geometry. Compared with the BDS-3/GPS dual-system PPP solution, the BDS-3/GPS/Galileo triple-system PPP solution reduces the horizontal convergence time by approximately 13.70–16.67% and the vertical convergence time by about 18.75–20.00% under cutoff angles from 7° to 30° based on the 68th percentile statistics. The 95th percentile results further confirm the advantage of the triple-system solution under a more stringent statistical criterion. Where convergence is achieved, the triple-system solution reduces the horizontal convergence time by approximately 6.0–7.3% and the vertical convergence time by about 15.3–26.0%. Moreover, the triple-system solution exhibits a smaller re-convergence jump under abnormal observation conditions. In addition, under high elevation cutoff conditions, the introduction of Galileo satellites effectively improves PPP availability, thereby enhancing the continuity and robustness of PPP. These results indicate that incorporating Galileo observation within the PPP-B2b framework can effectively improve PPP performance and provide a simple and practical approach for high-precision real-time positioning.

## 1. Introduction

The BeiDou Navigation Satellite System (BDS), as an important component of the global navigation satellite system (GNSS), provides continuous, all-weather, and high-precision positioning, navigation, and timing (PNT) services to users worldwide [1]. Since the proposing of Precise Point Positioning (PPP), high-precision single-receiver positioning has been achieved using precise satellite orbit and clock products. Compared with conventional differential positioning techniques, PPP can provide high-precision positioning services within a unified global reference frame [2,3,4,5]. However, the PPP accuracy largely depends on the quality of these precise products. In 2020, BDS-3 was fully completed and officially started providing global services. Meanwhile, the PPP-B2b service was launched as a distinctive feature of the system. Through three GEO satellites, corrections including satellite orbit corrections, satellite clock offset corrections and differential code biases are transmitted to users. Consequently, users can obtain real-time precise products without relying on ground communication networks, which significantly enhances its potential applications in communication-limited environments [6,7].

In view of these advantages, extensive studies have been conducted on the positioning performance based on PPP-B2b products. Existing research indicates that within China and surrounding regions, the PPP-B2b service is capable of providing stable real-time high-precision positioning, and its overall performance is comparable to that of mainstream real-time precise products [8,9,10,11]. In terms of applications, PPP-B2b has been validated in scenarios such as earthquake monitoring and marine positioning, demonstrating reliable real-time performance even in environments with limited or no communication links [12,13,14,15]. In recent years, research efforts have gradually shifted from performance evaluation of PPP-B2b products to improvements in service capability. To address GNSS signal blockage in complex environments, PPP-B2b has been integrated with inertial navigation systems (INS) to achieve continuous positioning during short outages, significantly improving positioning stability in urban areas [16,17]. However, the relatively long convergence time remains a key challenge for PPP-B2b, which is closely related to the accuracy of orbit and clock products. To mitigate residual orbit and clock offset errors, additional error modeling parameters have been introduced to enhance positioning accuracy [18]. To further improve convergence performance, previous studies have explored approaches such as ambiguity resolution and high-precision regional ionospheric modeling, both of which can effectively shorten convergence time in kinematic applications [19,20]. However, in service boundary areas, the positioning performance exhibits certain regional variations due to the availability of corrections and the number of visible satellites [21,22].

The current PPP-B2b service only broadcasts correction parameters for BDS-3 and GPS [1]. Some studies have attempted to combine the Galileo High Accuracy Service (HAS) products with PPP-B2b corrections, demonstrating that cross-system product integration can effectively improve positioning accuracy and convergence performance [23,24]. Related studies have also explored PPP approaches that combine HAS corrections with broadcast ephemeris for positioning, showing improved performance in challenging environments [25]. Nevertheless, such approaches typically require simultaneous access to multiple real-time precise correction products, which increases data acquisition requirements and system complexity, thus limiting their applicability in certain scenarios. In contrast, broadcast ephemeris can be directly decoded from satellite navigation messages without requiring any external communication links, making them a more autonomous and reliable alternative. Furthermore, with the continuous development of satellite navigation technologies, the accuracy of broadcast ephemeris has improved significantly in recent years. The global average root mean square (RMS) signal-in-space range error (SISRE) of the Galileo system has reached approximately 0.2 m, which is the smallest among the four global GNSS constellations [26]. This improvement is mainly attributed to the shorter update interval of navigation messages and the use of highly stable passive hydrogen maser clocks [27]. Although the broadcast ephemeris accuracy is lower than that of real-time precise products, previous studies have shown that its impact on positioning can be mitigated through appropriate error modeling and parameter estimation. Consequently, decimeter-level kinematic PPP accuracy can be achieved using broadcast ephemeris alone [28,29,30,31].

In this study, the impact of incorporating Galileo observation into the PPP-B2b framework on positioning performance is further investigated. As a regional augmentation system, PPP-B2b suffers from a limited number of visible satellites in service edge regions, which may degrade positioning performance for users. To address this limitation, Galileo observations based on broadcast ephemeris are introduced into the PPP-B2b framework, enabling multi-GNSS integration without relying on additional correction services. The remainder of this paper is organized as follows. First, the models and methods for precise product recovery and PPP are introduced. Then, with precise products as references, the accuracy of PPP-B2b corrections and broadcast ephemeris is evaluated. Subsequently, positioning experiments are conducted to assess the performance improvement of PPP-B2b positioning when Galileo observation is included. Finally, the main findings of this study are summarized.

## 2. Methods

### 2.1. PPP-B2b Product Recovery

The PPP-B2b service provides satellite corrections in state space representation. The satellite orbit corrections are expressed in the satellite-fixed coordinate system as radial (R), along-track (A), and cross-track (C) components. To recover the satellite position in the Earth-centered Earth-fixed (ECEF) frame, these corrections must first be transformed from the satellite-fixed frame to the terrestrial frame. The transformation is given by

(1) $$dX=\left[\begin{array}{ccc} e_{R} & e_{A} & e_{C} \end{array}\right]\left[\begin{array}{c} dR\\ dA\\ dC \end{array}\right]$$

where

(2) $$e_{R}=\frac{r}{|r|},e_{C}=\frac{r\times \dot{r}}{|r\times \dot{r}|},e_{A}=e_{C}\times e_{R}$$

where $\left[dR\;dA\;\;dC{]}^{T}\right.$ denotes the radial, along-track, and cross-track orbit corrections in the satellite-fixed frame; dX represents the transformed correction vector in the ECEF frame; $e_{R}$, $e_{A}$, and $e_{C}$ are the corresponding unit vectors; and r and $\dot{r}$ denote the satellite position and velocity vectors computed from broadcast ephemeris. Then, based on the satellite orbit calculated from broadcast ephemeris, the precise satellite orbit can be recovered as:

(3) $$X_{B2b}=X_{\mathrm{brdc}}-dX$$

where $X_{\mathrm{brdc}}$ and $X_{B2b}$ are satellite orbits obtained from the broadcast ephemeris and PPP-B2b service, respectively. Similarly, the precise satellite clock offset of PPP-B2b can be reconstructed from the broadcast satellite clock offset $dt_{\mathrm{brdc}}^{s}$ and the PPP-B2b clock offset correction parameter dClk as:

(4) $$dt_{B2b}^{s}=dt_{\mathrm{brdc}}^{s}-\frac{dClk}{c}$$

where c is the speed of light in vacuum.

### 2.2. PPP Model

In PPP processing, the dual-frequency ionosphere-free (IF) combination model is the most adopted observation model, which effectively eliminates the first-order ionospheric delay. Its simplified form is expressed as:

(5) $$\left\{\begin{array}{c} P_{IF}=\rho +c\left(dt_{r}-dt^{s}\right)+d_{trop}+\varepsilon_{P}\\ \phi_{IF}=\rho +c\;\left(dt_{r}-dt^{s}\right)+d_{trop}+N_{IF}+\varepsilon_{\phi } \end{array}\right.$$

where $P_{IF}$ and $\phi_{IF}$ denote the ionosphere-free combined pseudorange and carrier-phase observations, respectively. ρ represents the geometric distance between the receiver and satellite; $dt_{r}$ and $dt^{s}$ are the receiver clock offset and satellite clock offset, respectively; $d_{trop}$ denotes the tropospheric delay, which consists of the hydrostatic and wet components; $N_{IF}$ is the ambiguity term of the ionosphere-free combination; $\varepsilon_{P}$ and $\varepsilon_{\phi }$ represent the observation noises of pseudorange and carrier phase, respectively. It should be noted that the receiver and satellite clock offsets in the above model are not purely physical clock errors, since signal-dependent hardware delays can be absorbed into the clock terms. For Galileo broadcast ephemeris, the satellite clock offset is associated with the E1/E5a ionosphere-free combination [32]. For PPP-B2b products, the BDS-3 satellite clock offset is referenced to the B3I signal, whereas the GPS satellite clock offset is referenced to the L1/L2 ionosphere-free combination [33]. Thus, the Time Group Delay (TGD) parameter from Galileo broadcast ephemeris and code bias products from PPP-B2b products can be used to correct satellite code bias according to the signal types. After applying tropospheric delay corrections using empirical models, the residual wet component is typically treated as an unknown parameter and estimated in the PPP processing. The estimated parameters of the ionosphere-free PPP model include four types of parameters: three-dimensional receiver coordinates, receiver clock offset, tropospheric wet delay, and ionosphere-free ambiguity parameters:

(6) $$\left\{\begin{array}{c} E\left(l\right)=A\cdot {\left(\begin{array}{cccc} r & {dt}_{r} & ZWD & N_{IF} \end{array}\right)}^{T}\\ D\left(l\right)=\delta_{0}^{2}\left(\begin{array}{c} U_{2m}\\ 10^{-4}U_{2m} \end{array}\right) \end{array}\right.$$

(7) $$l={\left({\tilde{P}}_{r,IF}^{1},{\tilde{P}}_{r,IF}^{2},\cdots \;,{\tilde{P}}_{r,IF}^{m},{\tilde{\Phi }}_{r,IF}^{1},{\tilde{\Phi }}_{r,IF}^{2},\cdots \;,{\tilde{\Phi }}_{r,IF}^{m}\right)}^{T}$$

where A is the design matrix; r is the receiver position vector; $dt_{r}$ is the receiver clock offset parameter; ZWD is the zenith wet delay; $N_{IF}$ represents the ambiguity vector; $\delta_{0}^{2}$ is the variance factor of unit weight; $U_{2m}$ denotes a 2m×2m identity matrix; m is the number of observed satellites. The variance of pseudorange observations is set to be 100 times that of carrier-phase observations. The vector l represents the residuals of all observations from all visible satellites, i.e., the difference between the observed and computed values.

To realize PPP based on Broadcast Ephemeris (BCE), we introduce a compensation strategy for broadcast ephemeris errors based on the classical PPP model. Referring to the study of Carlin et al. [27], we adopt the simplest compensation approach. In this method, the unmodeled SISRE is absorbed by introducing an appropriate process noise to the float ambiguity parameters. Although this method is typically a simplified workaround compared to introducing SISRE-related parameters into the PPP model [30]. This approach does not require an extension of the state vector. Instead, it modifies the time-update step of the Kalman filter so that the ambiguities change from constant parameters in classical PPP to random-walk parameters. This allows the ambiguities to vary slowly over time, thereby partially compensating for the time-varying characteristics of SISRE.

To ensure that the model can be applied to both Precise Ephemeris (PRE) and Broadcast Ephemeris (BCE), we introduce a configurable process noise for the ionosphere-free ambiguity $N_{IF}$ within the Kalman filtering framework. In this way, the broadcast ephemeris errors can be compensated without modifying the structure of the state vector or the observation model. The state vector is uniformly expressed as

(8) $$x={\left[r\;\;dt_{r}\;\;ZWD\;\;N_{IF}\right]}^{T}$$

During the time-update step of the Kalman filter, the process noise covariance matrix $Q_{N_{IF}}$ for the ambiguity parameters is conditionally defined as:

(9) $$Q_{N_{IF}}=\left\{\begin{array}{cc} 0, & \mathrm{Precise}\;\mathrm{Ephemeris}\;\mathrm{mode}\;(\mathrm{PRE})\\ \sigma_{proc}^{2}, & \mathrm{Broadcast}\;\mathrm{Ephemeris}\;\mathrm{mode}\;(\mathrm{BCE}) \end{array}\right.$$

where $\sigma_{proc}$ denotes the standard deviation of the ambiguity process noise. The value of this process noise is set to 2 mm, and a detailed sensitivity analysis of the noise is presented in the next section.

### 2.3. PPP Availability

In theory, at least four satellites with valid observations are required at a given epoch for a station to complete a PPP solution; otherwise, the positioning result for that epoch will be unavailable. Introducing Galileo observation increases the number of satellites that can participate in the positioning solution, thereby improving the PPP availability when the number of available satellites is limited. To evaluate the impact of introducing Galileo observation on PPP availability, the following metric is used:

(10) $$C^{r_{i}}=\frac{N_{valid}}{N_{total}}\times 100\%$$

where $N_{valid}$ denotes the number of epochs with successful positioning solutions, $N_{total}$ denotes the total number of epochs, and $r_{i}$ represents the i-th station $\left(i=1,2,3,\ldots ,n\right)$. For all stations within a given day, the PPP availability $C^{r_{i}}$ is first calculated for each station. The daily average availability is then obtained by averaging the availability values of all stations:

(11) $$C^{DOY}=avg\left(C^{r_{1}},\cdots {,C}^{r_{n}}\right)$$

Furthermore, the average PPP availability among all the days is calculated by averaging the daily availability values:

(12) $$\bar{C}=avg\left(C^{DOY_{1}},\cdots {,C}^{DOY_{7}}\;\right)$$

where $\bar{C}$ is the PPP availability among all the days; avg(∗) is the averaging function.

## 3. Experiments

### 3.1. Data and Processing Strategy

**Figure 1:**
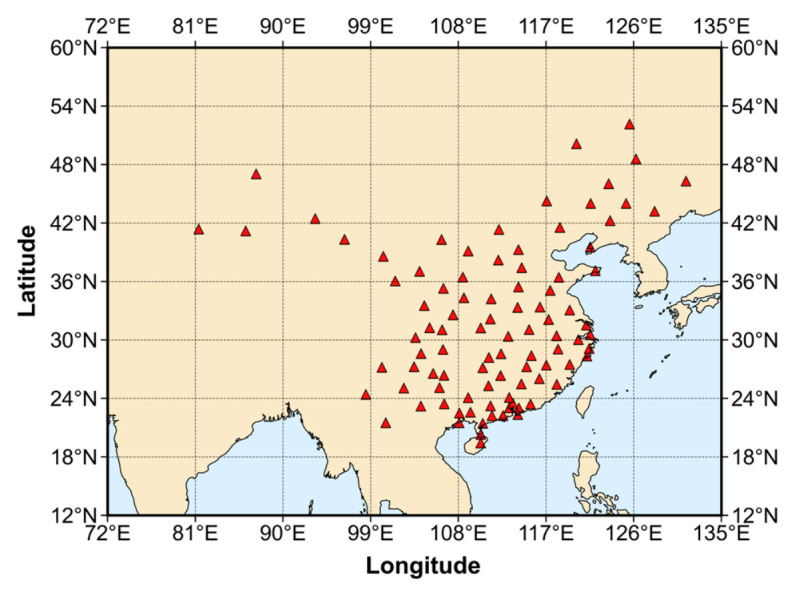
Distribution of PPP validation stations in China. Red triangles denote the locations of the PPP validation stations used in this study.

To analyze the performance improvement of PPP-B2b after incorporating broadcast ephemeris from the Galileo system, positioning experiments were conducted using data from 94 reference stations within the China region over a 7-day period from 22 March to 28 March 2024. The stations are established by a Chinese company, and their distribution is shown in Figure 1. The detailed PPP processing strategy is listed in Table 1. As for satellite code bias, GPS and Galileo do not require correction in this experiment. For BDS-3, it needs to convert the clock offset of B3I into the B1I/B3I ionosphere-free combination through its code bias products. To evaluate the impact of introducing Galileo observation into the PPP-B2b product, two positioning strategies were designed for comparison: (1) the original PPP-B2b product solution, and (2) the PPP-B2b solution integrated with Galileo observation. In addition, different elevation cutoff angles of 7°, 15°, 30°, and 45° were set to investigate positioning performance under different observation conditions, as well as PPP availability.

### 3.2. Product Performance Analysis

**Figure 2:**
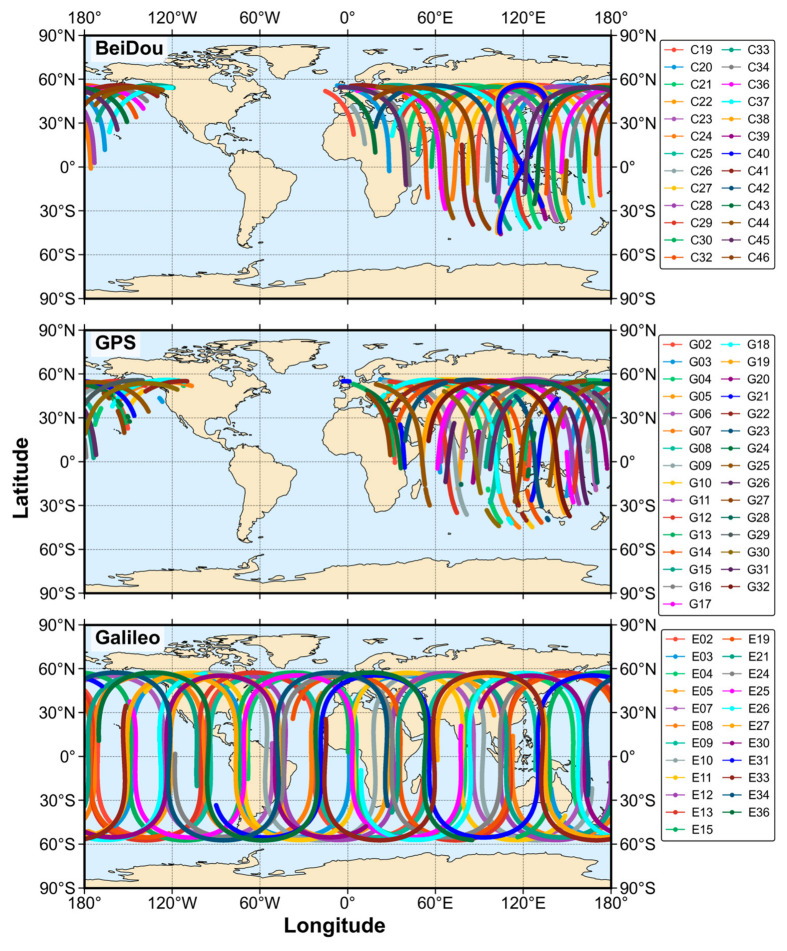
Sub-satellite ground tracks of BDS-3 and GPS satellites from the PPP-B2b product and Galileo satellites from the broadcast ephemeris.

Prior to the positioning experiments, the accuracy of PPP-B2b and Galileo broadcast products is evaluated with respect to post-processed precise products from the Center for Orbit Determination in Europe (CODE). The sub-satellite ground tracks of the BDS-3 and GPS satellites supported by the PPP-B2b product on 23 March 2024 (the second day of the selected experimental period) are shown in the first two subplots of Figure 2.

The ground tracks of the BDS-3 and GPS satellites included in the PPP-B2b product are largely confined to a triangular region approximately bounded by the three points (0° W, 60° N), (110° W, 60° N), and (100° E, 55° S). This region is consistent with the core service coverage of the PPP-B2b product. Also, the ground tracks of the introduced Galileo satellites are shown in the third subplot of Figure 2. Since Galileo satellites are introduced using broadcast ephemeris and are not included in the PPP-B2b correction framework, they are not constrained by the monitoring network. As a result, their sub-satellite tracks exhibit a more globally distributed pattern. As a complementary system, Galileo provides additional geometric strength, which can effectively increase the number of visible satellites and reduce the Dilution of Precision (DOP) values for users, particularly in edge regions of the service area or in dynamic positioning scenarios.

**Figure 3:**
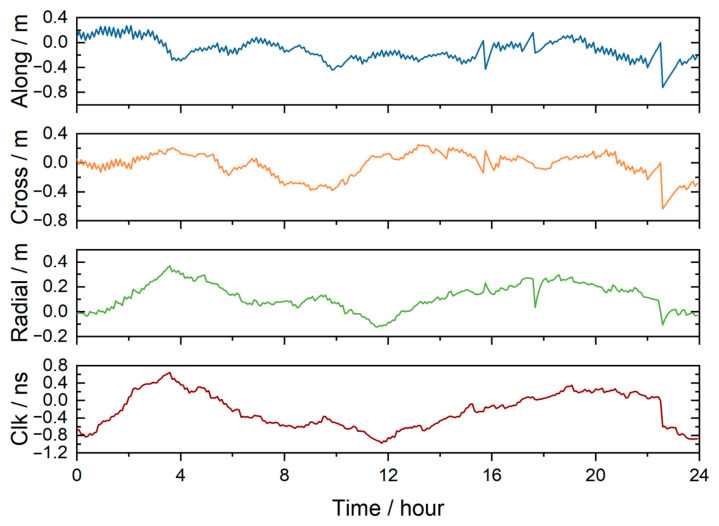
The satellite orbit and clock offset error time series of Galileo satellite E04 from the broadcast ephemeris on 23 March 2024.

Figure 3 presents the satellite orbit and clock offset error series of E04 on 23 March 2024. According to the figure, the along-track error magnitude remains within 0.75 m, the cross-track error within 0.65 m, and the radial error within 0.4 m, satisfying the decimeter-level accuracy requirement of broadcast ephemeris. The along-track component exhibits the most stable temporal variation, while the radial direction achieves the smallest RMS (0.16 m), indicating the highest orbit accuracy in that direction. The clock offset error ranges from −1.00 ns to 0.65 ns, corresponding to an equivalent range error of approximately −0.30 m to 0.20 m. The two exhibit similar temporal variations, indicating a strong coupling between orbit and clock accuracy.

**Figure 4:**
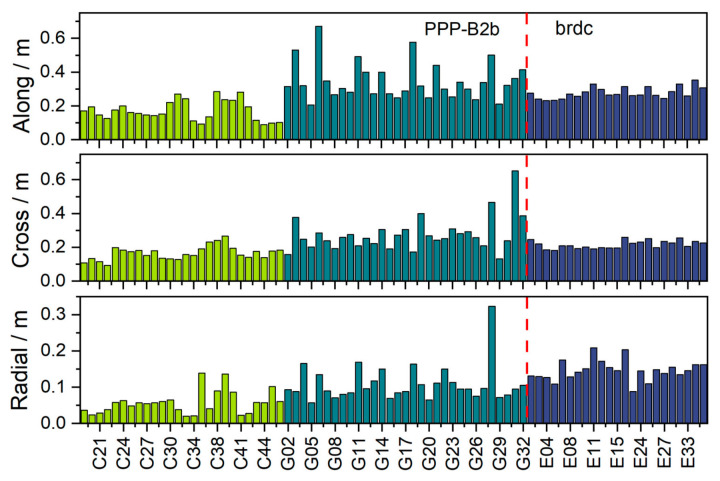
Orbit accuracy of BDS-3 and GPS satellites from the PPP-B2b product and Galileo satellites from the broadcast ephemeris product. Results of the three satellite systems are distinguished by different colors, and the red dashed line is used to separate the two types of products.

The seven-day mean RMS orbit error of each satellite was derived, as shown in Figure 4. In the along-track direction, the Galileo broadcast ephemeris achieves a mean RMS error of 0.28 m, which is lower than that of GPS but higher than that of BDS-3. The along-track performance of Galileo satellites is relatively stable across the constellation, whereas a few GPS satellites show noticeably larger errors. In the cross-track direction, Galileo satellites show a mean RMS error of 0.22 m, slightly worse than BDS-3 satellites but better than GPS satellites. In the radial direction, errors for all three systems are smaller than those in the along-track and cross-track components. The mean RMS error in the radial direction for Galileo satellites is 0.15 m, which is inferior to both BDS-3 and GPS satellites. BDS-3 demonstrates the best radial performance, with a system-wide average error of only 0.06 m. GPS also shows good radial performance overall, although satellite G28 exhibits a relatively large deviation.

**Figure 5:**
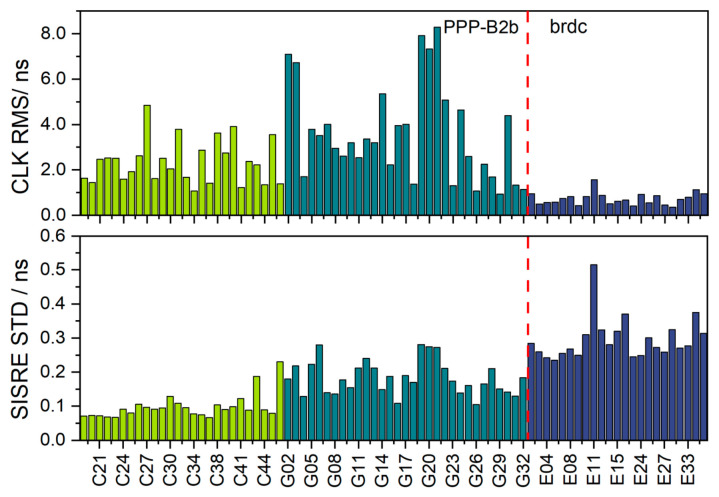
Clock offset accuracy and SISRE of BDS-3 and GPS satellites from the PPP-B2b product and Galileo satellites from the broadcast ephemeris product. Results of the three satellite systems are distinguished by different colors, and the red dashed line is used to separate the two types of products.

The resulting clock offset errors RMS statistics are presented in the first subplot of Figure 5. Because satellite orbit and clock offset errors are intrinsically coupled in their impact on user ranging performance, the signal-in-space range error (SISRE) is commonly adopted as a comprehensive metric for evaluating the combined quality of orbit and clock products. The second subplot of Figure 5 illustrates the seven-day average standard deviation (STD) of SISRE for each satellite.

The PPP-B2b clock accuracy obtained in this study is generally consistent with previously reported evaluation results, in which the clock offset errors RMS values were approximately 3 ns for GPS and 1.5 ns for BDS, with a clock offset STD of about 0.15 ns [9]. The relatively large RMS values of PPP-B2b clock products may be related to signal distortion biases (SDBs) and inconsistencies receiver types between PPP-B2b and IGS product estimation [34,35]. From the perspective of the seven-day mean clock offset RMS, Galileo broadcast ephemeris exhibits a smaller mean RMS clock offset error than the PPP-B2b satellite clock offset corrections, with a system-level average accuracy better than 0.75 ns. This result is consistent with expectations, as the PPP-B2b clock offset corrections are derived within a regional real-time framework and therefore may exhibit larger residual errors compared with broadcast clock products referenced to precise post-processing solutions. However, when evaluated using SISRE STD, Galileo satellites present a mean value of 0.30 ns, whereas BDS-3 and GPS satellites exhibit lower averages of 0.10 ns and 0.18 ns, respectively. The larger SISRE STD of Galileo broadcast products indicates comparatively lower ranging stability. In general, a smaller SISRE STD indicates more stable signal-in-space ranging performance and is beneficial to positioning applications. Therefore, from a comprehensive orbit–clock coupling perspective, the PPP-B2b-corrected BDS-3 and GPS satellites provide more stable orbit–clock quality for PPP user ranging.

**Figure 6:**
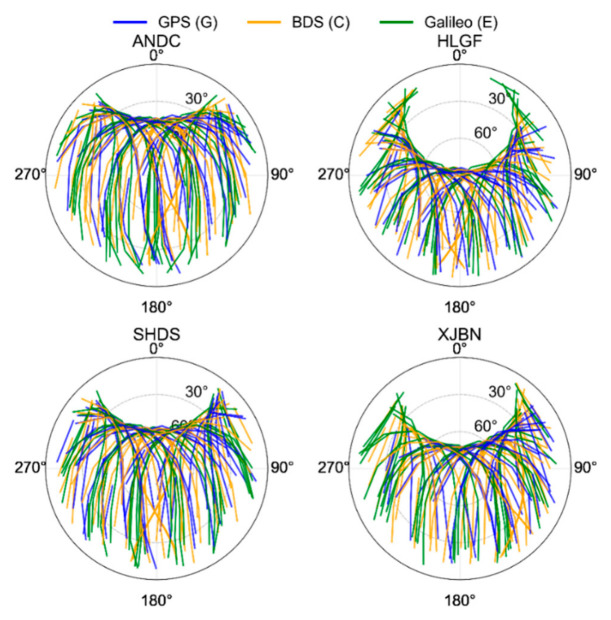
Skyplots of stations ANDC, HLGF, SHDS, and XJBN on 23 March 2024.

Figure 6 illustrates the 24-h skyplots for GPS, BDS-3, and Galileo satellites at stations ANDC, HLGF, SHDS, and XJBN on 23 March 2024. The satellite tracks at the four stations show a clear east–west distribution pattern, with azimuth angles mainly concentrated in the 90–270° range, while the north–south distribution is relatively limited. In terms of elevation, each station exhibits a certain number of high-elevation tracks, whereas low-elevation tracks are relatively scarce. As the elevation cutoff angle increases, the number of available low elevation observations decreases significantly, reducing geometric strength and thus being unfavorable for positioning. A comparison of the three-system tracks shows that Galileo satellites are relatively uniformly distributed across the four stations, forming effective coverage on both the eastern and western sides and filling gaps in some low-elevation directions, thereby making the overall sky coverage more balanced. The inclusion of Galileo improves the uniformity of satellite geometric distribution. This is consistent with the previously reported statistical results showing an increase in the number of visible satellites and a reduction in DOP, and also provides a geometric explanation for the convergence improvement observed under low and medium elevation cutoff angles.

**Figure 7:**
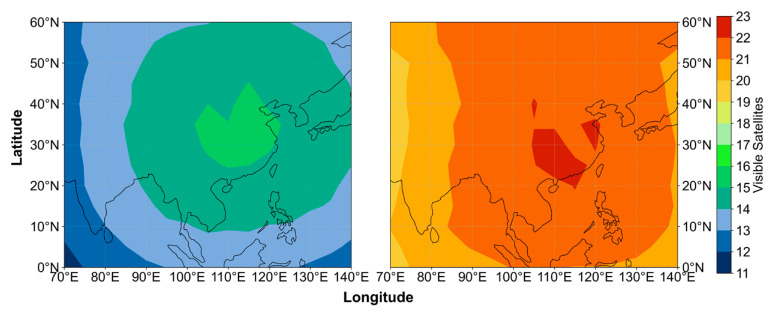
Visible satellite numbers under the PPP-B2b BDS-3+GPS dual-system solution (**left**) and after adding Galileo satellites (**right**).

**Figure 8:**
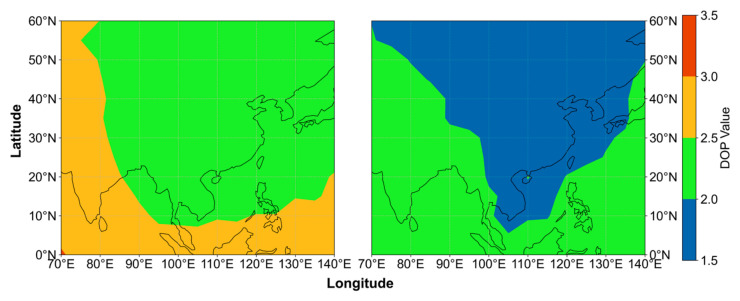
User DOP values under the PPP-B2b BDS-3+GPS dual-system solution (**left**) and after adding broadcast-ephemeris Galileo satellites (**right**).

To evaluate the distributions of visible satellite number and DOP in China and its surrounding regions, a 5° × 5° grid was established. At each grid point, satellite elevation angles were computed once per hour. A cutoff elevation angle of 7° was applied, and satellites with elevation angles greater than 7° were counted as visible. The corresponding DOP values were computed simultaneously. For each grid point, the 24-hourly results were averaged to obtain daily mean values, and the seven-day averages of both visible satellite number and DOP were subsequently derived. Two constellation configurations were analyzed: the dual-system configuration (BDS-3 + GPS) based on the PPP-B2b framework, and the triple-system configuration (BDS-3 + GPS + Galileo) incorporating broadcast ephemeris from Galileo. The distributions of visible satellite number and DOP under the two configurations are illustrated in Figure 7 and Figure 8.

Overall, the number of visible satellites is primarily influenced by constellation geometry and latitude. Under the dual-system configuration, the number of visible satellites in China and its surrounding regions generally exceeds 12, with a maximum of about 15 within China, and gradually decreases outward from the region. After the Galileo constellation is added, the number of visible satellites increases significantly, generally exceeding 19 within this area and reaching up to 22. The Galileo constellation effectively mitigates the geometric weakness of the dual-system configuration, leading to a notable improvement in satellite availability. This demonstrates that Galileo serves as an effective geometric complement to the regional PPP-B2b coverage.

DOP is a key factor affecting PPP accuracy and convergence time, quantifying the amplification effect of satellite geometry on positioning errors. Under identical measurement noise conditions, positioning errors are more sensitive to larger DOP values. In the original dual-system PPP-B2b configuration, the selected region lies within the service area. Therefore, the constellation geometry is favorable. The average DOP values in China and its surrounding regions are already relatively low, generally below 3.0. Within China and its adjacent areas, the values are even lower, falling below 2.5. After the Galileo constellation is added, DOP values decrease significantly. Most regions in China drop below 2.0, while the surrounding areas also decrease to below 2.5. These results indicate that, within the PPP-B2b based framework, adding Galileo significantly improves satellite geometry in the multi-GNSS positioning model. The improved geometric strength effectively reduces DOP and enhances the stability and reliability of PPP solutions.

### 3.3. Positioning Validation

Before the positioning experiment, a sensitivity analysis was first conducted to assess the influence of the ambiguity process noise on positioning performance. Based on the stations and processing strategy, three values of the ambiguity process noise, namely 1 mm, 2 mm, and 3 mm, were tested under an elevation cutoff angle of 7°. Two positioning cases were considered, including the Galileo-only solution using broadcast ephemeris and the BDS-3+GPS+Galileo triple-system solution. At each epoch, the 68th and 95th percentiles of the positioning errors from all stations were taken as representative positioning errors. For the Galileo-only solution, only the final positioning accuracy was analyzed because of its relatively slow convergence. For the triple-system solution, convergence was declared when the horizontal error was below 0.2 m and the vertical error was below 0.4 m, and the errors remained below these thresholds continuously for 5 min. The final positioning accuracy was also evaluated. The statistical results are listed in Table 2 and Table 3.

Table 2 presents the convergence time of the BDS-3+GPS+Galileo triple-system solution under different ambiguity process noise settings, based on the 68th and 95th percentile statistics. Under the 68th percentile statistic, the 1 mm setting shows the longest convergence time, whereas the 2 mm and 3 mm settings produce comparable convergence times. In contrast, under the 95th percentile statistic, the 1 mm setting leads to the shortest convergence time. Table 3 presents the convergence accuracy of the Galileo-only solution and the BDS-3+GPS+Galileo triple-system solution under different ambiguity process noise settings, also based on the 68th and 95th percentile statistics. Overall, the convergence accuracy of the CGE solution is not sensitive to the ambiguity process noise, with comparable accuracy obtained under the three tested settings. Therefore, the analysis focuses mainly on the convergence accuracy of the Galileo-only solution. For the Galileo-only solution, the effect of the process noise on positioning accuracy is at the millimeter level under both the 68th and 95th percentile statistics, indicating limited sensitivity. Nevertheless, the 2 mm setting provides relatively better positioning accuracy. Considering the results in Table 2 and Table 3, 2 mm was selected as the value of the ambiguity process noise $\sigma_{\mathrm{proc}}$ for the subsequent experiments.

To evaluate the improvement achieved by adding Galileo to the PPP-B2b based framework, a comparative PPP experiment was conducted based on the stations and processing strategy. Observations collected on 23 March 2024 at station AXSA, located in central China, were selected. Different elevation cutoff angles (7°, 15°, 30° and 45°) were considered. Two processing schemes were implemented: the BDS-3+GPS PPP solution (CG) and the BDS-3+GPS+Galileo PPP solution (CGE). A reconvergence interval of 120 min was imposed in the experiment.

**Figure 9:**
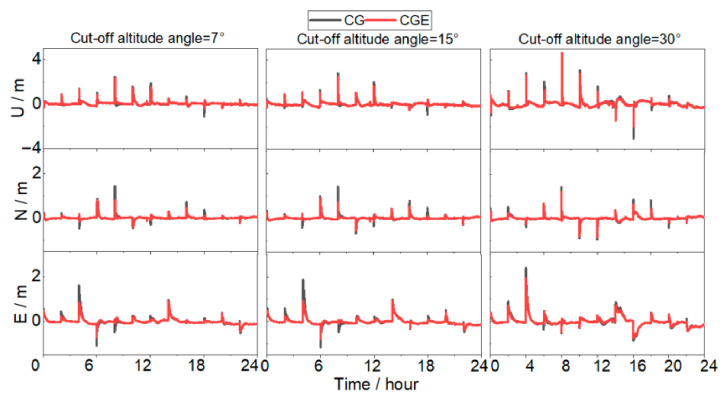
Positioning error series in the U, N, and E directions at station AXSA on 23 March 2024.

Figure 9 presents the 24-h positioning error time series in the Up (U), North (N), and East (E) components under the three elevation cutoff angles. At a 7° cutoff angle, sufficient visible satellites and favorable satellite geometry result in comparable overall error levels for both CG and CGE solutions. The amplitude differences among the three components are small. The CGE solution exhibits slightly reduced peak errors during certain periods, with a smoother time series overall. When the cutoff angle increases to 15°, low-elevation satellites are excluded, reducing observational redundancy. Under this condition, the dual-system CG solution shows more pronounced oscillations in the vertical (U) component. In contrast, the CGE solution demonstrates improved stability, with noticeably smaller peak amplitudes. This indicates that the inclusion of Galileo satellites strengthens the observation geometry and mitigates the increase in DOP caused by the higher cutoff angle. At a 30° cutoff angle, geometric degradation becomes more severe. The CG solution exhibits large fluctuations in all three components, particularly in the vertical direction, where peak errors increase significantly. Although the CGE solution is also affected by the restricted observation geometry, its error amplitudes remain consistently smaller than those of the CG solution, and the time series continuity is better preserved, demonstrating stronger resistance to geometric deterioration.

**Figure 10:**
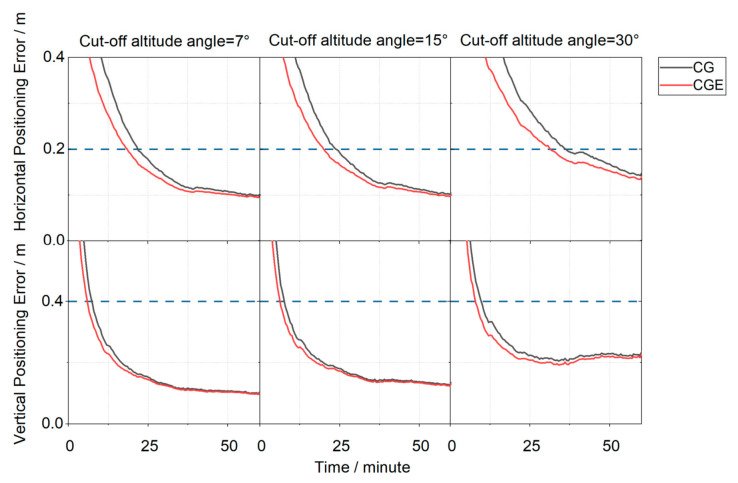
Positioning error convergence series for all stations based on the 68th percentile.

**Figure 11:**
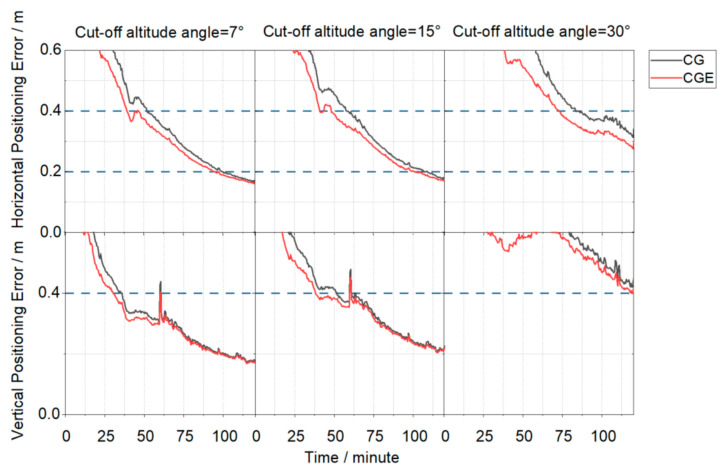
Positioning error convergence series for all stations based on the 95th percentile.

Figure 10 and Figure 11 present the positioning error time series aggregated over all stations under three elevation cutoff angles, comparing the CG dual-system and CGE triple-system positioning strategies. In Figure 10, the positioning errors from all stations are collected at each epoch, and the 68th percentile of these errors is taken as the representative positioning error for that epoch. Convergence is declared when the horizontal error is below 0.2 m and the vertical error is below 0.4 m, and the error must remain below these thresholds continuously for 5 min. Under the 7° elevation cutoff angle, the horizontal convergence time of the CG dual-system solution is 22 min, while the CGE triple-system, after introducing Galileo observation, achieves convergence in 18.5 min, corresponding to a 15.91% reduction in convergence time. In the vertical direction, the convergence time decreases from 7.5 min for the CG solution to 6 min for the CGE solution, representing a 20.00% reduction. When the elevation cutoff angle increases to 15°, the CGE triple-system reduces the convergence time by 16.67% in the horizontal direction and 18.75% in the vertical direction compared with the CG dual-system solution. Under the 30° elevation cutoff angle, the convergence time of the CGE solution is reduced by 13.70% horizontally and 20.00% vertically. Under the 68th percentile statistic, the CGE solution achieves shorter convergence times than the CG solution. From the perspective of convergence time reduction ratios, the improvement achieved by the triple-system solution over the dual-system solution remains relatively stable across different elevation cutoff angles. Under lower elevation cutoff angles, the inclusion of Galileo satellites introduces observations from more diverse spatial directions. This would theoretically strengthen the satellite geometry. However, the dual-system geometry is already sufficiently robust, so the additional improvement becomes marginal. The reduction in horizontal convergence time is approximately 13.70–16.67%, while that in the vertical direction is about 18.75–20.00%. This indicates that the introduction of Galileo observation leads to a more significant improvement in vertical convergence performance. This can be attributed to the fact that the additional satellites enhance the satellite geometry, thereby providing a more pronounced improvement in the vertical component, which is generally more sensitive to geometric weakness.

Figure 11 shows the positioning error convergence curves for all stations based on the 95th percentile. Under the 95th percentile statistic, the required convergence time increases significantly. Under this metric, the CGE triple-system solution still outperforms the CG dual-system solution. At a 7° elevation cutoff angle, the CGE solution reduces the horizontal convergence time by 6.0% and the vertical convergence time by 15.3% compared to the CG solution. When the cutoff angle increases to 15°, the reductions are 7.3% in the horizontal direction and 26.0% in the vertical direction. At a 30° elevation cutoff angle, neither solution converges to the predefined thresholds (horizontal ≤ 0.2 m and vertical ≤ 0.4 m) within the experimental period, while the CGE solution still achieves better final positioning accuracy than the CG solution. It should be noted that a reconvergence jump caused by abnormal observations occurs in the vertical direction at t = 60 min. Taking the 7° cutoff angle as an example, the vertical error of the CG solution increases from 0.31 m to 0.43 m, whereas that of the CGE solution increases from 0.30 m to 0.37 m. The jump magnitude of the CGE solution is reduced by approximately 40% compared to that of the CG solution, indicating that the inclusion of Galileo observations enhances the robustness of the filter against observation anomalies and enables a faster recovery during reconvergence. Overall, under the more stringent 95th percentile statistic, the inclusion of Galileo observations not only improves convergence performance but also enhances the stability and robustness of the positioning results under abnormal observation conditions.

**Figure 12:**
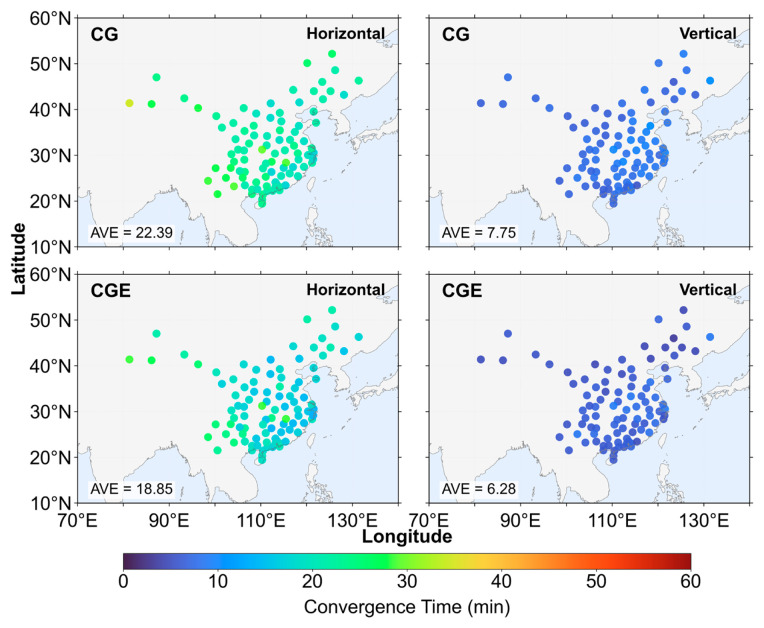
Positioning convergence time at 7° elevation cutoff angle.

**Figure 13:**
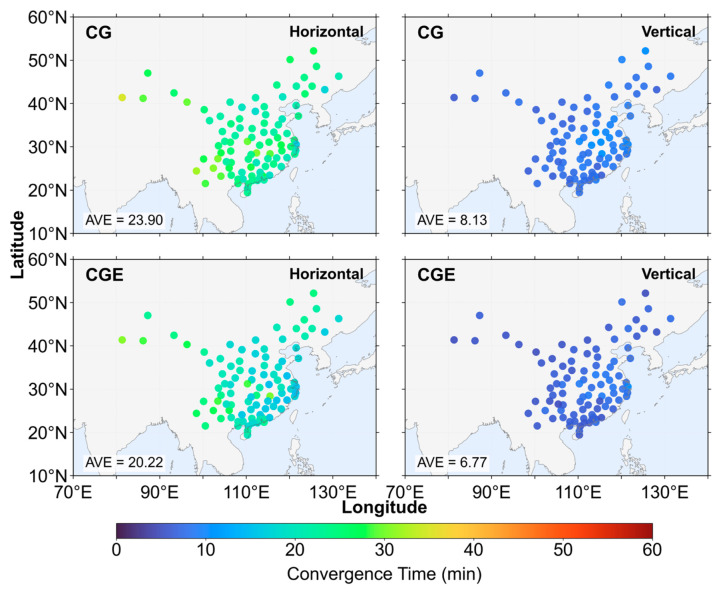
Positioning convergence time at 15° elevation cutoff angle.

**Figure 14:**
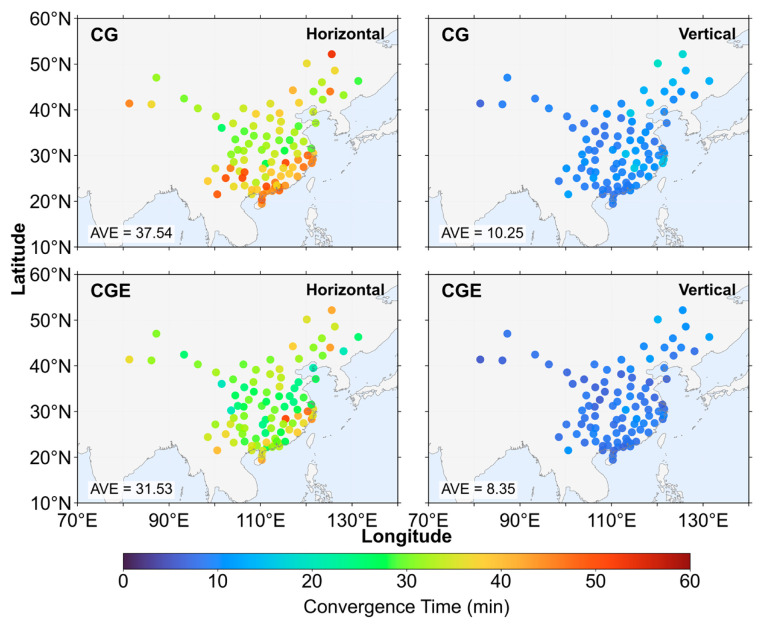
Positioning convergence time at 30° elevation cutoff angle.

Figure 12, Figure 13 and Figure 14 present the distribution of positioning convergence times for all stations under three elevation cutoff angles. Overall, the average convergence times under the 7° and 15° cutoff angles are very similar, indicating that within this range the change in cutoff angle has a limited impact on convergence performance, and the observation geometry remains relatively stable. However, when the cutoff angle increases to 30°, the positioning convergence performance degrades significantly. Compared with the 15° cutoff angle, the average horizontal convergence time increases by approximately 11–13 min, corresponding to an increase of 55.93–57.07%, while the average vertical convergence time increases by about 1.58–2.12 min, corresponding to an increase of 23.33–26.08%. This indicates that under higher elevation cutoff conditions, the horizontal convergence performance is more significantly affected. Under the 7° cutoff angle, the reduction in horizontal convergence time is mainly concentrated at stations located in the central and southern regions of China, whereas the improvement in the vertical direction does not exhibit a clear spatial distribution pattern. At the 15° cutoff angle, the spatial distribution of convergence improvement is generally consistent with that under the 7° condition. However, in the horizontal direction, several stations in the southeastern region show more pronounced reductions in convergence time. In contrast, under the 30° cutoff angle, the improvement achieved by the three-system strategy becomes more evident. In the horizontal direction, the convergence time is reduced for stations located in the northeastern marginal region of China, while more significant convergence improvements are observed for most stations in the southern region. In the vertical direction, stations along the eastern coastal region also exhibit relatively concentrated and noticeable improvements.

Table 4 presents the seven-day average PPP availability under four elevation cutoff angles (7°, 15°, 30°, and 45°) for both the CG strategy and the CGE strategy, as well as the improvement in availability achieved by the CGE strategy relative to the CG strategy. The results show that when the elevation cutoff angle does not exceed 30°, the PPP availability of the CG strategy is already close to 100%, indicating that sufficient satellites are available for positioning under these conditions. Consequently, the introduction of Galileo observation produces only a marginal improvement in PPP availability. However, when the cutoff angle increases to 45°, the PPP availability of the CG dual-system solution decreases significantly, dropping by 14.8% compared with the 30° cutoff angle. At this point, the contribution of Galileo satellites becomes evident, increasing the PPP availability to 96.8%. The introduction of Galileo observation improves PPP availability, indicating that Galileo satellites can alleviate the shortage of available satellites under high elevation cutoff angles and thereby enhance the reliability of positioning solutions.

## 4. Conclusions

Nowadays, real-time precise positioning has become increasingly important in applications such as autonomous navigation, disaster monitoring, and precision agriculture. The BeiDou PPP-B2b service can provide decimeter-level positioning without relying on ground networks. However, its performance in challenging environments is limited by the number of visible satellites and satellite geometry since it currently supports only BDS-3 and GPS. Meanwhile, the Galileo system has achieved significant improvements in its broadcast ephemeris accuracy, offering an opportunity to enhance multi-GNSS PPP. Thus, we propose a method for enhancing the positioning performance of PPP-B2b by integrating Galileo observation. The method was validated by using observations from 94 stations in China collected between 22 March and 28 March 2024.

First, the accuracy of PPP-B2b products and Galileo broadcast ephemeris was assessed. The results show that the Galileo broadcast ephemeris achieve decimeter-level orbit accuracy, with mean RMS errors of 0.28 m, 0.22 m, and 0.15 m in the along-track, cross-track, and radial directions, respectively, while the mean RMS clock offset error is better than 0.75 ns. Although the Galileo SISRE STD is larger than those of the PPP-B2b products, the introduction of Galileo observations significantly increases the number of visible satellites and reduces DOP values, leading to faster convergence. This suggests that the improvement in convergence performance is primarily driven by enhanced satellite geometry in the initial stage, whereas the final positioning accuracy is more strongly influenced by orbit and clock offset error quality.

Second, PPP experiments were conducted under different elevation cutoff angles to compare the convergence performance of the dual-system and triple-system strategies. Based on 68th percentile statistics, convergence was defined as the epoch when the horizontal error fell below 0.2 m and the vertical error fell below 0.4 m, and remained below these thresholds for 5 consecutive minutes. The results show that introducing Galileo observation consistently improves convergence performance. Compared with the dual-system PPP, the triple-system PPP reduces the horizontal convergence time by approximately 13.70–16.67% and the vertical convergence time by about 18.75–20.00% under different cutoff angles. The results based on the 95th percentile statistics further show that the triple-system PPP still outperforms the dual-system PPP under a more stringent statistical criterion, reducing the horizontal convergence time by approximately 6.0–7.3% and the vertical convergence time by about 15.3–26.0%. In addition, the triple-system PPP shows a smaller reconvergence jump under abnormal observation conditions, indicating improved robustness and recovery capability. At the 30° cutoff angle, neither solution reaches the predefined convergence thresholds within the experimental period under the 95th percentile statistics, while the triple-system PPP still achieves better final positioning accuracy. Further spatial analysis shows that under 7° and 15° cutoff angles, the improvements in convergence performance are mainly concentrated in the central and southern regions of China. When the cutoff angle increases to 30°, the improvements become more evident in the southern and northeastern marginal regions, while the eastern coastal regions show noticeable improvements in the vertical component.

Finally, the PPP availability under different cutoff angles was evaluated. When the cutoff angle is less than 30°, the PPP availability of the dual-system PPP is close to 100%, indicating that sufficient satellites are available for positioning and that the improvement brought by Galileo satellites is limited. However, when the cutoff angle increases to 45°, the PPP availability of the dual-system PPP decreases significantly, whereas the triple-system PPP shows a noticeable improvement. High cutoff angles typically correspond to challenging environments such as urban canyons, dense building areas, or mountainous regions, where satellite visibility is limited. Under such conditions, the introduction of Galileo satellites can increase the number of usable satellites and improve the continuity and robustness of PPP.

In summary, introducing Galileo observation can effectively improve PPP-B2b positioning performance by increasing satellite availability and enhancing satellite geometry, with particularly significant benefits under complex observation environments. Considering that broadcast ephemeris can be directly decoded from satellite navigation messages without requiring external communication links, this study adopts broadcast products to maintain a fully autonomous PPP-B2b processing framework. It is noted that Galileo High Accuracy Service (HAS), as a free global service, provides more precise orbit and clock offset corrections than broadcast ephemeris. The integration of HAS may further improve positioning performance, which warrants further investigation.

## Figures and Tables

**Table 1:** PPP processing strategy.

Troposphere	Strategies	
	Dual-System Solution	Triple-System Solution
Data period	22 March 2024–28 March 2024	
GNSS	BDS-3+GPS	BDS-3+GPS+Galileo
Orbit and clock products	PPP-B2b products	BDS-3 + GPS: PPP-B2b products Galileo: broadcast ephemeris
Data sampling interval	30 s	
Satellite elevation cutoff angle	7°/15°/30°/45°	
Ionosphere	Ionosphere-free linear combination	
Troposphere	GPT2 model	
Ambiguity strategy	BDS-3/GPS: float solution, estimated as a constant within each observation arc Galileo: float solution with process noise considered	
Observation weighting scheme	Elevation angle-dependent weighting method $\sigma^{2}=\left\{\begin{array}{c} 1.0,\;elev\geq 10{}^{\circ}\\ 1/(2.3662*\sin(elev)),elev<10{}^{\circ} \end{array}\right.$	
Satellite PCO/PCV corrections	Corrected using igs14.atx file	
Filtering method	Square Root Information Filter	

**Table 2:** Convergence time of the BDS-3+GPS+Galileo triple-system solution based on the 68th and 95th percentile statistics.

Positioning Strategy and Statistical Method	Convergence Time (min) Under Different Ambiguity Process Noise Settings		
	1 mm	2 mm	3 mm
CGE 68th	20.0	18.5	18.5
CGE 95th	90.0	93.5	97.5

**Table 3:** Positioning accuracy of the Galileo-only solution and the BDS-3+GPS+Galileo triple-system solution based on the 68th and 95th percentile statistics.

Positioning Strategy and Statistical Method		Positioning Accuracy (m) Under Different Ambiguity Process Noise Settings		
		1 mm	2 mm	3 mm
E 68th	Horizontal	0.31	0.31	0.33
	Vertical	0.43	0.42	0.44
E 95th	Horizontal	0.71	0.70	0.72
	Vertical	1.05	1.01	1.02
CGE 68th	Horizontal	0.07	0.07	0.07
	Vertical	0.07	0.07	0.07
CGE 95th	Horizontal	0.16	0.16	0.16
	Vertical	0.17	0.17	0.17

**Table 4:** Seven-day average PPP availability under the two positioning strategies.

Strategy	PPP Availability (%) Under Different Elevation Cutoff Angles			
	7°	15°	30°	45°
CG	99.8	99.8	99.8	85.0
CGE	99.8	99.8	99.8	96.8
Improvement	0.0	0.0	0.0	11.8

## Data Availability

Dataset available on request from the authors.

## References

[1] China Satellite Navigation Office (2019). BeiDou Navigation Satellite System Development Report (Version 4.0).

[2] Malys S., Jensen P.A. (1990). Geodetic Point Positioning with GPS Carrier Beat Phase Data from the CASA UNO Experiment. Geophys. Res. Lett..

[3] Zumberge J.F., Heflin M.B., Jefferson D.C., Watkins M.M., Webb F.H. (1997). Precise Point Positioning for the Efficient and Robust Analysis of GPS Data from Large Networks. J. Geophys. Res. Solid Earth.

[4] Kouba J., Héroux P. (2001). Precise Point Positioning Using IGS Orbit and Clock Products. GPS Solut..

[5] Kouba J., Lahaye F., Tétreault P., Teunissen P.J.G., Montenbruck O. (2017). Precise Point Positioning. Springer Handbook of Global Navigation Satellite Systems.

[6] China Satellite Navigation Office (2020). PPP-B2b Precise Point Positioning Service Signal—Space Signal Interface Control Document (Version 1.0).

[7] Xu Y., Yang Y., Li J. (2021). Performance Evaluation of BDS-3 PPP-B2b Precise Point Positioning Service. GPS Solut..

[8] Zhang W., Lou Y., Song W., Sun W., Zou X., Gong X. (2022). Initial Assessment of BDS-3 Precise Point Positioning Service on GEO B2b Signal. Adv. Space Res..

[9] Tao J., Liu J., Hu Z., Zhao Q., Chen G., Ju B. (2021). Initial Assessment of the BDS-3 PPP-B2b RTS Compared with the CNES RTS. GPS Solut..

[10] Nie Z., Xu X., Wang Z., Du J. (2021). Initial Assessment of BDS PPP-B2b Service: Precision of Orbit and Clock Corrections, and PPP Performance. Remote Sens..

[11] Ren Z., Gong H., Peng J., Tang C., Huang X., Sun G. (2021). Performance Assessment of Real-Time Precise Point Positioning Using BDS PPP-B2b Service Signal. Adv. Space Res..

[12] Yang H., Ji S., Weng D., Wang Z., He K., Chen W. (2021). Assessment of the Feasibility of PPP-B2b Service for Real-Time Coseismic Displacement Retrieval. Remote Sens..

[13] Geng T., Li Z., Xie X., Liu W., Li Y., Zhao Q. (2022). Real-Time Ocean Precise Point Positioning with BDS-3 Service Signal PPP-B2b. Measurement.

[14] Yu D., Li H., Ji B., Chen Y., Huang Y., Li X., Wang Z. (2025). Analysis of Marine Real-Time PPP Accuracy with the BDS-3 PPP-B2b Service. Comput. Electr. Eng..

[15] Zang J., Fan S., Xu C., Li Z., Fang R., Lou Y. (2024). Performance Assessment of the BDS-3 PPP-B2b Service for Real-Time Earthquake Source Description: A Case Study for the 2021 Mw 7.4 Maduo Earthquake. GPS Solut..

[16] Lai L., Meng X., Zhao D., Li X., Guo W., Li L. (2023). PPP/INS Tight Integration with BDS−3 PPP−B2b Service in the Urban Environment. Sensors.

[17] Xu X., Nie Z., Wang Z., Wang B., Du Q. (2022). Performance Assessment of BDS-3 PPP-B2b/INS Loosely Coupled Integration. Remote Sens..

[18] Xu X., Nie Z., Wang Z., Zhang Y., Dong L. (2023). An Improved BDS-3 PPP-B2b Positioning Approach by Estimating Signal in Space Range Errors. GPS Solut..

[19] Yuan L., Li B., Miao W., Ge H., Wu Z. (2025). PPP-B2b-RTK: A PPP-B2b Augmentation Method by Using the SSR Corrections from a Single Reference Station. GPS Solut..

[20] Zhao Q., Pan S., Gao W., Tao X., Liu H., Zhang Z., Wang Q. (2025). Enhancing PPP-B2b Performance with Regional Atmospheric Augmentation. Remote Sens..

[21] Liu Y., Yang C., Zhang M. (2022). Comprehensive Analyses of PPP-B2b Performance in China and Surrounding Areas. Remote Sens..

[22] Zhou H., Fu W., Wang L., Li T., Wu Y., Chen R., Li J. (2023). Multi-Frequency BDS-3 Real-Time Positioning Performance Assessment Using New PPP-B2b Augmentation Service. IEEE Sens. J..

[23] Wei H., Xiao G., Zhou P., Li P., Xiao Z., Zhang B. (2025). Combining Galileo HAS and Beidou PPP-B2b with Helmert Coordinate Transformation Method. GPS Solut..

[24] Zheng K., Ye Z., Zhao J., Ma Q., Fu W., Hu J., Liu K., Tang L., Zhang X. (2026). Real-Time PPP with the Fusion of Galileo HAS and BDS-3 PPP-B2b. Measurement.

[25] Yi D., Naciri N., Bisnath S. (2024). Precise Positioning Utilizing Smartphone GNSS/IMU Integration with the Combination of Galileo High Accuracy Service (HAS) Corrections and Broadcast Ephemerides. GPS Solut..

[26] Montenbruck O., Steigenberger P., Hauschild A. (2018). Multi-GNSS Signal-in-Space Range Error Assessment—Methodology and Results. Adv. Space Res..

[27] Carlin L., Hauschild A., Montenbruck O. (2021). Precise Point Positioning with GPS and Galileo Broadcast Ephemerides. GPS Solut..

[28] Wang F., Gong X., Sang J., Zhang X. (2015). A Novel Method for Precise Onboard Real-Time Orbit Determination with a Standalone GPS Receiver. Sensors.

[29] Montenbruck O., Ramos-Bosch P. (2008). Precision Real-Time Navigation of LEO Satellites Using Global Positioning System Measurements. GPS Solut..

[30] Gunning K., Blanch J., Walter T. SBAS Corrections for PPP Integrity with Solution Separation. Proceedings of the 2019 International Technical Meeting of the Institute of Navigation.

[31] Guo R., Zhang X., Gu S., Peng Y., Gong X., Lou Y. (2026). Improving PPP Performance through Satellite Clock Drift Fitting Based on Broadcast Ephemeris. Measurement.

[32] Ge Y., Zhou F., Sun B., Wang S., Shi B. (2017). The Impact of Satellite Time Group Delay and Inter-Frequency Differential Code Bias Corrections on Multi-GNSS Combined Positioning. Sensors.

[33] Dai W., Liu N., Zhang Z., Tang C., Zhang Z., Yang Y., Pan L. (2025). BDS-3/GPS Uncombined Real-Time PPP with PPP-B2b Precise Products: Modeling and Its Long-Term Performance Evaluation. Adv. Space Res..

[34] Hauschild A., Steigenberger P., Montenbruck O. Inter-Receiver GNSS Pseudorange Biases and Their Effect on Clock and DCB Estimation. Proceedings of the 32nd International Technical Meeting of the Satellite Division of The Institute of Navigation (ION GNSS+ 2019).

[35] Gong X., Zheng F., Gu S., Zhang Z., Lou Y. (2022). The Long-Term Characteristics of GNSS Signal Distortion Biases and Their Empirical Corrections. GPS Solut..

